# Serine protease inhibitors of the whirling disease parasite *Myxobolus cerebralis* (Cnidaria, Myxozoa): Expression profiling and functional predictions

**DOI:** 10.1371/journal.pone.0249266

**Published:** 2021-03-29

**Authors:** Edit Eszterbauer, Dóra Szegő, Krisztina Ursu, Dóra Sipos, Ákos Gellért

**Affiliations:** 1 Institute for Veterinary Medical Research, Centre for Agricultural Research, Budapest, Hungary; 2 Veterinary Diagnostic Directorate, National Food Chain Safety Office, Budapest, Hungary; INRS, CANADA

## Abstract

Here, we studied the expression pattern and putative function of four, previously identified serine protease inhibitors (serpins) of *Myxobolus cerebralis*, a pathogenic myxozoan species (Cnidaria: Myxozoa) causing whirling disease of salmonid fishes. The relative expression profiles of serpins were determined at different developmental stages both in fish and in annelid hosts using serpin-specific qPCR assays. The expression of serpin Mc-S1 was similar throughout the life cycle, whereas a significant decrease was detected in the relative expression of Mc-S3 and Mc-S5 during the development in fish, and then in the sporogonic stage in the worm host. A decreasing tendency could also be observed in the expression of Mc-S4 in fish, which was, however, upregulated in the worm host. For the first time, we predicted the function of *M*. *cerebralis* serpins by the use of several bioinformatics-based applications. Mc-S1 is putatively a chymotrypsin-like inhibitor that locates extracellularly and is capable of heparin binding. The other three serpins are caspase-like inhibitors, and they are probably involved in protease and cell degradation processes during the early stage of fish invasion.

## Introduction

Myxozoans are obligate endoparasites belonging to the phylum Cnidaria that includes mainly free-living species, such as sea anemones, hydras or jellyfish. The body organization of myxozoans is simple, and the two remarkably different spore types (i.e. myxospores and actinospores) are characterized by richness in form [1, 2]. The two-host life cycle involving a vertebrate (mainly fish) and an invertebrate host (annelids or bryozoans) makes them a challenging parasite group to study [3]. Despite their taxonomic divergence and their evolution to parasitism [4, 5], myxozoans retain nematocysts (called polar capsules) that are structurally and functionally homologous to those of their free-living cnidarian relatives [6]. Although most myxozoans are harmless, some of the species affect the health of both farmed and wild fish populations, causing diseases and mortality. Despite their impact, effective protection against these parasites is not yet available [3]. A widespread pathogenic species is *Myxobolus cerebralis* which is responsible for whirling disease and causes declines among susceptible salmonid fry [7–9]. Both its intrapiscine and intraoligochaete development has been studied in detail [10, 11], and special attention has been paid to host susceptibility [12–17]. Although our knowledge on the genetic background of host–parasite interaction of *M*. *cerebralis* is scarce, some crucial moments of its development have been studied in this context. Previous studies revealed that the serine and cathepsin Z-like proteases of *M*. *cerebralis* were upregulated in the gills of fish during the first few hours of intrapiscine development [18, 19]. Similarly, the upregulation of parasite genes related to motility, cell division and cytoskeleton functioning was detected during fish recognition and subsequent invasion of sporoplasms of *M*. *cerebralis* actinospores (called triactinomyxon, TAM) [20].

Serine protease inhibitors (serpins) are a widely distributed superfamily of proteins with various biological functions [21]. Serpins are suicide inhibitors; they are cleaved by the target protease at the P1 position of the reactive center loop (RCL), which causes irreversible conformational changes and leads to inactivation of the serpin [22]. Proteases and their inhibitors have been reported as factors important for the invasion and immune evasion of parasites [23, 24]. Schistosome serpins of the blood fluke *Schistosoma mansoni* are involved in the post-translational regulation of schistosome-derived proteases as well as in parasite defense mechanisms against the action of host proteases [25]. Studies have revealed that the serpins of nematodes interact with endogenous parasite proteinases, counteract digestion by host proteinases, inhibit the host immune response, or even may act as immunomodulators [26–28]. Thereby serpins might be promising targets for the development of antiparasitic therapies. Serpins have also been detected in myxozoan parasites. Genomic and transcriptomic studies revealed that 19 protease inhibitors were putatively secreted by the virulent myxozoan parasite, *Thelohanellus kitauei*. Most of them were serine or cysteine protease inhibitors, which may regulate the activity of such host proteases [29]. The serpins in the genome of *T*. *kitauei* extensively diversified compared to those of the free-living cnidarians or other myxozoans [29, 30]. The recently elucidated myxozoan transcriptomes, such as those of *Henneguya salminicola* [31], *Ceratonova shasta* [32] or *Sphaerospora molnari* [33], have made it possible to identify new myxozoan serpin homologs, and to study their genetic diversity in detail [30]. Among 224 serpins from 71 species ranging from protists to vertebrates (including ten myxozoan species), seven *M*. *cerebralis* serpins (named Mc-S1 to Mc-S7) were distinguished, which formed four clusters on the phylogenetic tree. The entire coding region sequences of the serpins Mc-S1, Mc-S3, Mc-S4 and Mc-S5 (one of each phylogenetic cluster) have been identified successfully [30].

In the present study, the expression profile of the above four *M*. *cerebralis* serpins was determined at different stages of development both in fish and in annelid hosts. The coding region of serpins was characterized and compared. The protein structures were modeled *in silico*, and special attention was given to the RCL region. The functional analysis involved the prediction of inhibitory activity and secretion.

## Materials and methods

### Source of hosts and parasites

The myxozoan parasite *M*. *cerebralis* used for the exposure trials originated from the life cycles maintained in the laboratory of the Institute for Veterinary Medical Research, Budapest, Hungary since 2007, as described by Eszterbauer et al. [7]. The parasite spores (both myxospores and actinospores) were regularly checked by microscopy and DNA sequencing following the protocol by Sipos et al. [14], to exclude any possible contamination. Contaminant myxozoans were not detected in any case.

Rainbow trout *Onchorhynchus mykiss* (Kamloops strain) were obtained from the Lillafüred Trout Hatchery in Miskolc-Lillafüred, Hungary (48°6′59.22"N, 20°34′ 46.21"E). Trout fry were kept in a parasite-free environment in the hatchery, and transported to the laboratory approximately a week after hatching, as ‘yolk-sac stage’ larvae. Prior to exposure, the fish were reared in aerated, water flow-through aquaria at 15°C, until the age of 2 months.

### Experimental exposure

Parasite TAMs were harvested by filtering the water from the culture containers through 20 μm nylon mesh. TAMs used for the infection trials were less than 2 days old [7]. Two months old, naïve rainbow trout fry (average size 3 cm in length) were exposed individually in 100 ml de-chlorinated tap water at 15°C for 2 hrs. Non-exposed control fish were kept under the same conditions with the exception of the addition of TAMs. After exposure, fish were transferred into aquaria and separated by treatment group. Twenty exposed fish were sampled at 2 hrs (i.e. fish invasion stage; IP-2h), 2 days (i.e. early intrapiscine stage; IP-2d), respectively, and ten fish at 90 days (i.e. late intrapiscine/sporogonic stage; IP-90d) post exposure (p.e.). On the basis of previous findings [13], low infection intensities and aggregated parasite distribution were predicted in the early developmental stages. Thus we expected that the amount of parasite RNA might not reach the detection limit of the developed qPCR assays in every case. Therefore, we doubled the number of samples (20 vs. 10) for the early, IP-2h and IP-2d stages. The dose of exposure was 12,000 TAMs/fish for early-stage sampling (IP-2h and IP-2d), whereas 5,000 TAMs/fish for IP-90d sampling (to avoid fish mortality in sporogonic stage due to parasite overload). Prior to sampling at 2 hrs p.e., fish were placed in parasite-free tap water for 2–3 minutes to remove TAMs, which might have attached to the fish skin but have not penetrated the skin or gills. Then the fish were anesthetized with 200 mg/ml MS222 (Sigma), and killed with the application of a cervical cut. According to the developmental pathway of the parasite [10], skin (approximately 1 cm^2^) and gills (two gill arches) were collected 2 hrs and 2 days p.e., whereas fish heads without gills and eyes were sampled 90 days p.e.. Samples were fixed in RNAlater (Sigma) at +4°C overnight, and then stored at -20°C until subsequent molecular use.

The National Scientific Ethics Committee on Animal Experimentation provided approval for the animal experiments (No. PEI/001/4087-4/2015). Animal welfare was a priority, especially while handling experimental fish. Fish were kept under laboratory conditions. To maintain proper oxygen levels in glass tanks, the water was aerated constantly. Appropriate water quality was achieved using biological filters and continuous water flow-through. Fish were fed *ad libitum* with commercially available trout pellet (Aller Aqua) twice a day. Glass tanks were lightly illuminated for 12 hrs a day, and partial shielding and hiding tubes were provided to fish. The well-being of fish was monitored twice a day. None of the fish died during the exposure and incubation period.

For studying the expression of serpin genes in the intraoligochaete (IO) developmental stage, specimens of the annelid host *Tubifex tubifex* were exposed individually in 2-ml microtubes, as described by Marton & Eszterbauer [34]. The homogenized and concentrated *M*. *cerebralis* myxospore suspension was mixed with autoclaved mud. Worms were placed one by one in a 2-ml tube with 1.5 ml mud containing 10^3^–10^4^ myxospores. The rack with tubes was placed in a 5-l aerated plastic box filled with dechlorinated tap water. Prior to sampling 90 days p.e. (IO-90d), each worm was individually placed in 24-cell-well-plates in 1 ml de-chlorinated tap water for one day, to allow the worms to empty their gut content [35]. The water in the wells was checked for the presence of TAMs. Ten exposed worms (half of which released TAMs) were sampled and fixed as a whole in RNAlater.

### Molecular methods

Tissue samples stored in RNAlater were homogenized in liquid nitrogen using sterile, RNase-free, ceramic mortar and pestle. Up to 40 mg homogenized tissue per sample was used for total RNA extraction with RNeasy Mini Kit (Qiagen) according to the manufacturer’s instructions. In-column DNase treatment was performed with RNase-Free DNase Set (Qiagen). Extracted total RNA was stabilized with 16 U Protector RNase Inhibitor (Roche). The amounts of RNA and cDNA were measured with Qubit 3.0 Fluorometer system (Thermo Fisher Scientific, Life Technologies). In average, 500 ng total RNA per sample was used for cDNA synthesis with QuantiTect Reverse Transcription kit (Qiagen), following the manufacturer’s guidelines. Three samples (one from groups IP-2h, IP-90d and IO-90d, respectively), the total RNA concentration of which was below 25 ng/μl, were excluded from further examinations. Quantitative real-time PCR (qPCR) was used to detect the expression profiles of four serpin (i.e. target) genes, Mc-S1, Mc-S3, Mc-S4 and Mc-S5 of *M*. *cerebralis*. The primers and Taqman probes (listed in Table 1) were designed based on the coding region of serpins sequenced previously by Eszterbauer et al. [30]. Two reference genes, glyceraldehyde 3-phosphate dehydrogenase (GAPDH) and 60S ribosomal protein L18 (RPL18) of *M*. *cerebralis*, were applied for normalization. The qPCR assays were developed and/or optimized in the present study. GAPDH was run in a multiplex reaction with the target genes, respectively, whereas RPL18 was detected in a single qPCR assay.

**Table 1:** Oligonucleotides and Taqman probes used for *Myxobolus cerebralis* serpin-specific qPCR assays.

	Gene	Primer name	Primer sequence (5’ → 3’)	Probe name	Probe sequence (5’ → 3’)	Amplicon size (bp)	GenBank reference	Reference
**Reference**	GAPDH	McGAPDH_854F	GTGGCAAAACCCGCAACTAA	McGAPDH_883P	/5HEX/TGATAACGC/ZEN/AATCAAGGCTGCT /3IABkFQ/	95	GBKL01017634	Kosakyan et al. [55] probe: present study
		McGAPDH_948R	TGTGCGTCGACAAACTGGAT					
	RPL18	McRPL18_252F	CCAAAGCTGGAAATGATGCAAA	McRPL18P	/56-FAM/AGTAGTGGT/ ZEN/TGGCTCGATCCTCGA/3IABkFQ/	77	GBKL01047522; GBKL01049125	present study
		McRPL18_327R	TGGGATAGTATGGTAACGGTCAT					
**Target**	Mc-S1	McSerp1_896F	GTGATTTGAGCAATATGTCTGATGAG	McS1_955P	/56-FAM/ TCGCAGAAT /ZEN/GTATTACGTCACTGAC/3IABkFQ/	87	MT701001	present study
		McSerp1_982R	CAACACCTTCTTCGTTGATTTCG					
	Mc-S3	McSerp3_255F	GTCAAACAATTCAAAGGATCTCGTG	McS3_283P	/56-FAM/ CGATTGGAG /ZEN/TCGTTATTAACAAGCA /3IABkFQ/	78	MT701003	primers: Eszterbauer et al. [30]; probe: present study
		McSerp3_332R	AAGAAATATTCTGTCGACCCTTTCA					
	Mc-S4	McSerp4_845F	GGAGTGTTTGGAGAGCATGAAA	McS4_876P	/56-FAM/CTGTTTACT/ ZEN/CCTTACGTATCAGATTTGTCC/3IABkFQ/	87	MT701002	forward primer: Eszterbauer et al. [30]; reverse primer & probe: present study
		McSerp4_931R	ACCGATGATTGAGTATCCGAGA					
	Mc-S5	McSerp5_398F	AGCCATTGGAAACAGATACG	McS5_480P	/56-FAM/CGAACTGTC /ZEN/AAAATGATCTTCCC /3IABkFQ/	112	MT701004	primers: Eszterbauer et al. [30]; probe: present study
		McSerp5_509R	GAGATGTAAAACGTCTCAAGCATAG					

Reference genes were glyceraldehyde-3-phosphate dehydrogenase (GAPDH) and ribosomal protein L18 (RPL18). Probes labeled with HEX and FAM 5’ fluorochromes, respectively, ZEN internal quencher and a 3’ dark quencher (Iowa Black FQ; 3IABkFQ).

The qPCRs were performed using the QuantiTect Probe PCR kit (Qiagen) in 25 μl total volume. The reactions were composed of 2 μl (45–88 ng) template cDNA, 1× QuantiTect Probe PCR Master Mix including 4 mM MgCl_2_, 400 nM primers and 120 nM Taqman probes, except for GAPDH, where 200 nM primers and 60 nM Taqman probe were used. The reactions were run on a Rotor-Gene 6000 real-time cycler (Qiagen). The amplification started with initial denaturation at 95°C for 15 min, followed by 40 cycles of two steps, first at 95°C for 10 sec and then at 60°C for 60 sec. Ten-fold dilution series of gBlock Gene Fragments (synthesized by IDT) of target and reference genes (with calculated copy numbers of target DNA) were used for preparing standard curves, in triplicate reactions. The baselines and thresholds were manually set following visual examination of every run. Raw Cq (threshold cycle) values were calculated with the software Rotor-Gene Q version 2.3.1 (Qiagen). Cq values were not recorded when the curves did not reach the threshold value after 40 cycles. The descriptive values (slope, *y* intercept, coefficient of determination–R^2, etc.) of all qPCR runs are listed in S1A and S1B Tables. The lowest copy number of genes was 100, which was detectable with reliability in every run. The normalization of raw Cq data to the reference genes, and the inter-run calibration was performed with the software qBasePlus [36] to eliminate the effect of amplification efficiency differences among qPCR runs [37, 38]. Calibrated normalized relative quantities (CNRQ values) were calculated, which represented the relative amount of the target (serpin) genes compared to the amount of reference genes. Calculation was performed in two ways: once with normalizing data to both housekeeping genes, GAPDH and RPL18, and as a second (alternative) option, using only GAPDH as reference gene.

### Statistical analysis

Prior to statistical analyses, CNRQ values were log10 transformed to obtain normal distribution. Statistical analyses were performed using the R program, version 3.5.1 [39]. The group-level differences were tested with ANOVA, and considered significant at *p* < 0.05. As the assumptions of ANOVA were fullfilled, Tukey’s post hoc-test was applied to compare groups (i.e. developmental stages) with one another.

### *In silico* protein modeling

For structure modeling, the entire amino acid (aa) sequence of the coding region of the examined four serpins was available in GenBank: Mc-S1 (accession No. MT701001), Mc-S3 (MT701003), Mc-S4 (MT701002) and Mc-S5 (MT701004) [30].

The I-TASSER automatic protein structure modeling server [40] was used to generate the models of *M*. *cerebralis* serpins (Mc-S1, Mc-S3, Mc-S4 and Mc-S5) to predict the latent (closed) and the active (native) serpin conformations with reliable confidence scores (C-score) at the same time. The modeling server I-TASSER automatically selected the best template structures (PDB IDs: 1IZ2, 2VDX, 1E03, 4KDS and 5CDZ) to thread the serpin models. Pairwise protein sequence alignments among the examined serpins and their templates were calculated with the EMBOSS Needle tool [41]. The raw protein model structures were refined with the MacroModel energy minimization module of the Schrödinger Suite [42] to eliminate the steric conflicts between the side-chain atoms. We used PROCHECK [43] to calculate the Ramachandran plots, and two independent protein structure quality evaluation methods, PROSESS [44] and ProSA [45], were used to characterize the models. These Global Structure Assessment scores were deduced from other structure quality indicators such as overall quality, covalent and geometric quality, non-covalent/packing quality and torsion angle quality. Electrostatic potential maps were calculated with Adaptive Poisson–Boltzmann Solver (APBS) version 1.3 [46] using the linearized Poisson–Boltzmann method [46] with a dielectric constant of 78.0 and 2 for the water solvent and the protein core, respectively. The partial charges in the electrostatic potential were calculated with PDB2PQR [46]. Molecular graphics were created with VMD version 1.9.3 [47].

### Bioinformatics in modeling

Structure-based aa sequence alignment and the secondary structure prediction of the closed serpin forms were prepared using the Multiple Sequence Viewer module of Schrödinger Maestro. Secretion predictions were carried out using OutCyte [48], SecretomeP 2.0 [49], and SRTpred [50]. The RCL region was analyzed, and cleavage sites were compared using ExPASy Peptide Cutter database [51] to detect the possible protease targets for each serpin examined. Biological functions were predicted using COFACTOR [52].

To check the heparin binding affinity of serpins, the ClusPro 2.0 protein docking server was used in heparin docking mode [53]. The closed (latent) serpin model structures were used as receptor, while a small heparin chain (tetramer) was used as a ligand provided by the ClusPro heparin docking setup panel. The docking calculations were run in restrained mode where the helix D of each serpin was set as attractive region.

For RCL peptide binding characterization of the selected proteases, human chymotrypsin C (PDB ID: 4H4F), human cathepsin G (PDB ID: 1CGH), and caspase-3 from zebrafish (PDB ID: 5JFT), the Glide docking module of Schrödinger Suite was used [54]. The docking grid centers were set to the P1 side chain binding pockets of each protease, and the edge of the docking grid boxes was 30 Å. The octamer RCL peptide fragments (P4–P4’) were prepared using the LigPrep application of Schrödinger, which generated a conformation library for all RCL peptide octamers. Then, the default settings of the Standard Precision (SP) protocol of Glide were used for docking the ligand libraries into the target protease P1 binding sites.

## Results

### Experimental exposure and infection prevalence

In total, the relative expression profile of four *M*. *cerebralis* serpin genes was studied on 57 of 60 samples collected from exposed hosts at four different time points. Due to the insufficient amount of total RNA obtained, three samples were excluded from the qPCR analysis, thus the expression levels of serpins were estimated in 19 of 20 samples for developmental stage IP-2h, 9 of 10 for IP-90d, and 9 of 10 for IO-90d. The overall prevalence of infection was 100% based on the qPCR result of the housekeeping gene, GAPDH. Most likely due to the lower efficiency of RPL18 qPCR assay (S1 Table), five samples, which were positive to GAPDH, gave negative results for RPL18 (S2 Table).

### Relative expression profiling

**Fig 1:**
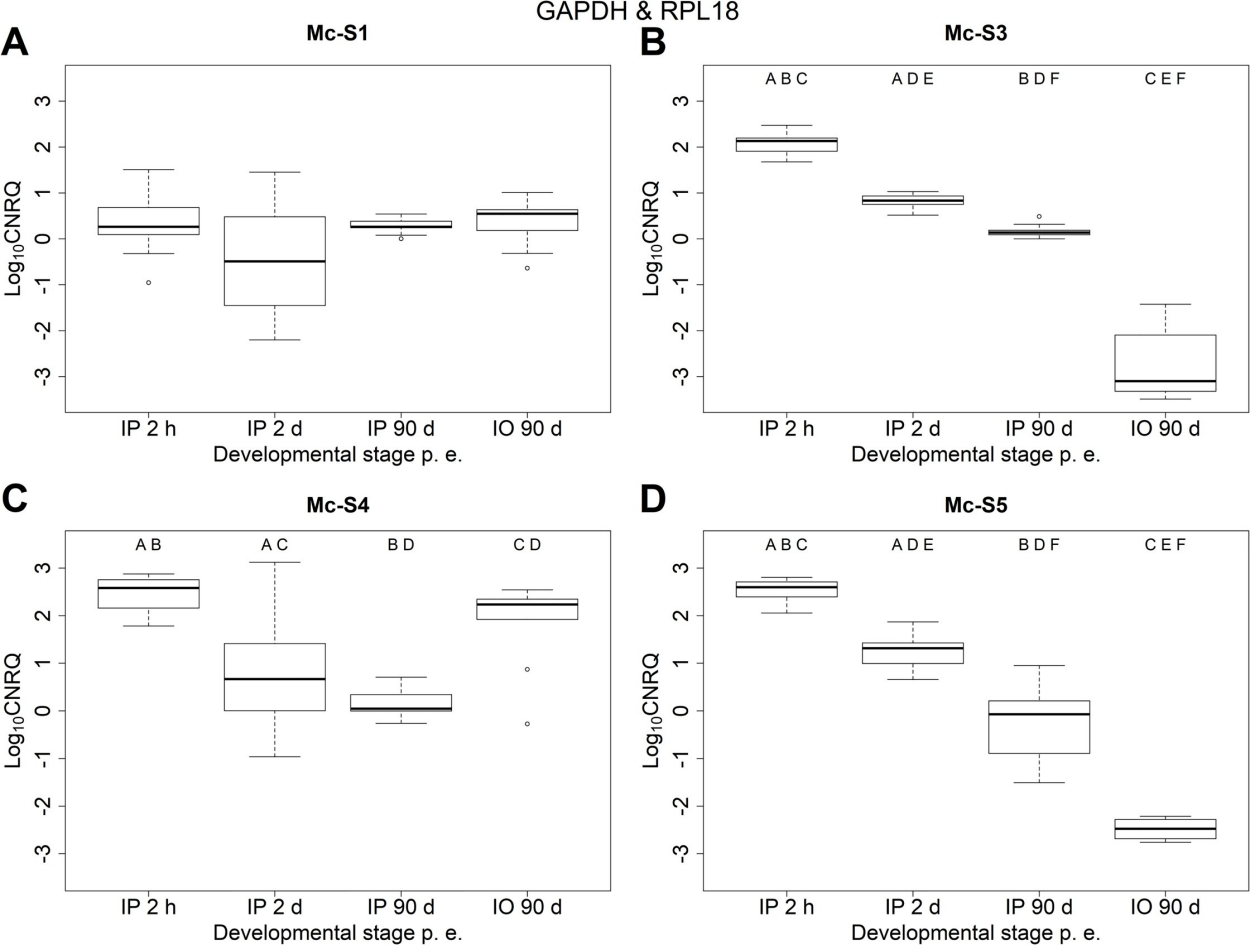
Relative expressions of serpin genes (A) Mc-S1, (B) Mc-S3, (C) Mc-S4, (D) Mc-S5 at different time points of *Myxobolus cerebralis* development. Log10 transformed, calibrated normalized relative quantity (log10 CNRQ) values were normalized to reference genes GAPDH and RPL18. IP: intrapiscine, IO: intraoligochaete developmental stage. Boxes represent the interquartile range between first and third quartiles. Bold line in the box is the median. Significant differences indicated with uppercase letters (*p* < 0.001). The same letter was given when the difference was significant between two groups.

The expression profiles of the four serpins examined were different when data were normalized to both GAPDH and RPL18 qPCR values (Fig 1, Tables 2 and 3). ANOVA showed significant differences in the relative expression of all four serpins (Table 3). The relative expression level of Mc-S1 was similar at all stages of development. The differences were not significant among any of the examined groups (i.e. developmental stages). For Mc-S3 and Mc-S5, the relative expression levels significantly decreased over time, and the lowest values were measured in the worm host (IO-90d). The expression of Mc-S4 significantly decreased in the early IP development, but then in the later stage (IP-90d) its expression remained at a similar level as in IP-2d. The highest expression level was detected in the worm host, although the difference between IP-2h and IO-90d was not significant.

**Table 2:** Descriptive statistics of the relative expression of *Myxobolus cerebralis* serpins (Mc-S1, Mc-S3, Mc-S4 and Mc-S5) in four different developmental stages on the basis of log-transformed, calibrated, normalized, relative quantities (Log10CNRQ).

Serpin name	Developmental stage	log_10_CNRQ			N_+_/N_t_
		Min–Max	Median	SD	
Mc-S1	IP 2 h p.e.	-0.9543–1.5083	0.2627	0.6524	12/19
	IP 2 d p.e.	-2.1987–1.4530	-0.4904	1.4614	5/20
	IP 90 d p.e.	0.0000–0.5405	0.2641	0.1686	9/9
	IO 90 d p.e.	-0.6407–1.0095	0.5468	0.5371	9/9
Mc-S3	IP 2 h p.e.	1.6768–2.4729	2.1319	0.2070	14/19
	IP 2 d p.e.	0.5168–1.0306	0.8314	0.1203	20/20
	IP 90 d p.e.	0.0000–0.4876	0.1383	0.1511	9/9
	IO 90 d p.e.	-3.4888 –-1.4230	-3.0989	0.8130	6/9
Mc-S4	IP 2 h p.e.	1.7826–2.8763	2.5817	0.3623	14/19
	IP 2 d p.e.	-0.9630–3.1196	0.6688	1.0900	16/20
	IP 90 d p.e.	-0.2621–0.7084	0.0449	0.2920	9/9
	IO 90 d p.e.	-0.2755–2.5411	2.2330	0.9334	9/9
Mc-S5	IP 2 h p.e.	2.0531–2.8020	2.5967	0.2364	14/19
	IP 2 d p.e.	0.6572–1.8673	1.3154	0.3247	20/20
	IP 90 d p.e.	-1.5081–0.9522	-0.0715	0.7913	8/9
	IO 90 d p.e.	-2.7605 –-2.2146	-2.4755	0.2470	4/9

Values were normalized to GAPDH and RPL18 reference genes. Minimum and maximum intensity (Min–Max); standard deviation (SD); number of positive/total samples (N+/Nt). IP: intrapiscine, IO: intraoligochaete developmental stage.

**Table 3:** *P*-values of ANOVA and Tukey’s post-hoc tests on log-transformed, calibrated, normalized, relative quantities (Log10CNRQ) obtained with measuring the relative expression of serpins (Mc-S1, Mc-S3, Mc-S4 and Mc-S5) in four different developmental stages of *Myxobolus cerebralis*.

Tukey’s test	Mc-S1	Mc-S3	Mc-S4	Mc-S5
IP 2 d–IP 2 h	–	<1e-04	< 0.001	<1e-09
IP 90 d–IP 2 h	–	<1e-04	< 0.001	<1e-09
IO 90 d–IP 2 h	–	<1e-04	0.25517	<1e-09
IP 90 d–IP 2 d	–	<1e-04	0.37328	<1e-09
IO 90 d–IP 2 d	–	<1e-04	0.00602	<1e-09
IO 90 d–IP 90 d	–	<1e-04	< 0.001	<1e-09
ANOVA	0.1785	< 2.2e-16	1.295e-08	< 2.2e-16

Reference genes: GAPDH and RPL18. IP: intrapiscine, IO: intraoligochaete developmental stage.

Alternatively, when only GAPDH values were used for normalization, the tendencies in the changes of expression levels were similar to the results obtained with two reference genes (S1 Fig, S3 Table in S1 File). Nevertheless, more data were obtained (as qPCR on RPL18 gave negative results in five cases), and statistical analysis resulted in a stronger support among some of the groups, especially for Mc-S1 (S4 Table in S1 File).

#### *In silico* models and structure comparison

**Fig 2:**
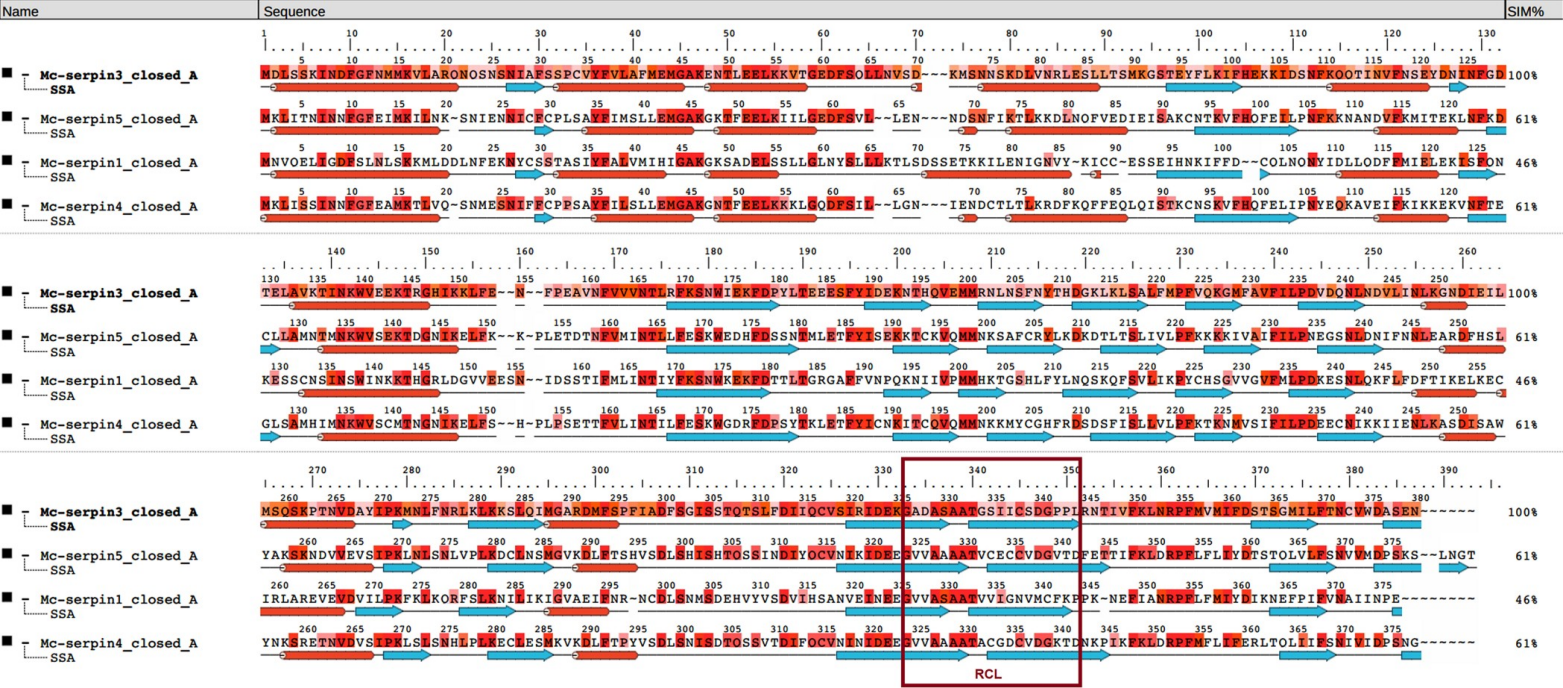
Structure-based amino acid sequence alignment of the closed form of *Myxobolus cerebralis* serpins Mc-S1, Mc-S3, Mc-S4 and Mc-S5. RCL: reactive center loop.

**Fig 3:**
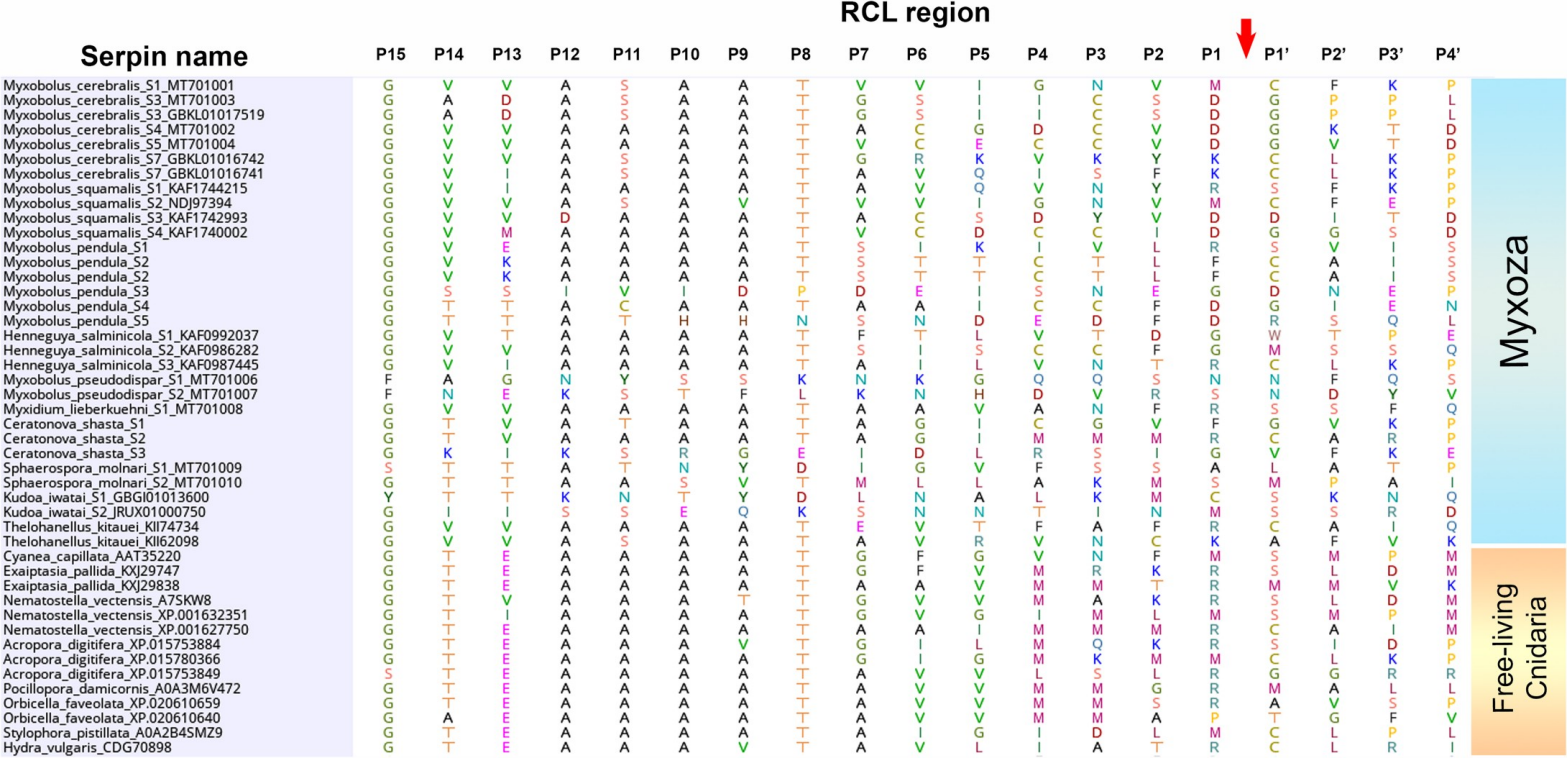
Amino acid sequences of the reactive center loop (RCL) region in the serpins of myxozoans and free-living cnidarians. The cleavage site of target protease is labeled with an arrow.

The structure-based aa sequence analysis confirmed that all examined proteins have regular serpin-structure with nine α-helices and three β-sheets (Fig 2). The relevant structural elements such as helices C, D, F, and the putative heparin-binding site were identified in all serpins (Fig 2, Table 4). The functionally most crucial part, the RCL, was composed of 16 aa in all serpins. At the cleavage site in the RCL region, three serpins (Mc-S3, Mc-S4 and Mc-S5) had Asp(D)–Gly(G) in P1–P1’ position, whereas for Mc-S1, Met(M)–Cys(C) were detected. Furthermore, high Ala content was observed in P12–P9 positions. As a common feature of serpins, notable structural and sequence-based differences were found among the RCL of myxozoans (Fig 3). The majority of the *M*. *cerebralis* serpins (4 out of 7) had Asp in position P1, and surprisingly, RCL was rich in Cys in some cases (e.g. Mc-S4 and Mc-S5). The presence of Asp in position P1 was not common in other myxozoans, although it was detected in some of the *M*. *squamalis* and *M*. *pendula* serpins (Fig 3). Most of the known serpins of free-living cnidarians have Arg(R) and Met(M) in P1 (Fig 3).

**Table 4:** Relevant amino acid (aa) positions in the examined serpins of *Myxobolus cerebralis*.

	Mc-S1	Mc-S3	Mc-S4	Mc-S5
**RCL** (reactive center loop)	326–344	326–344	324–342	324–342
**RCL (P15–P4’) sequence**	GVVASAATVVIGNVMCFKP	GADASAATGSIICSDGPPL	GVVAAAATACGDCVDGKTD	GVVAAAATVCECCVDGVTD
**RCL (P4–P4’) sequence**	GNVM-CFKP	ICSD-GPPL	DCVD-GKTD	CCVD-GVTD
**helix C**	48–8	48–58	47–57	47–57
**helix D** (heparin binding site)	71–87	68–84	66–82	68–82
**loop after hD**	88–94	84–90	83–87	83–90
**helix F**	129–142	130–143	127–141	127–141

**Fig 4:**
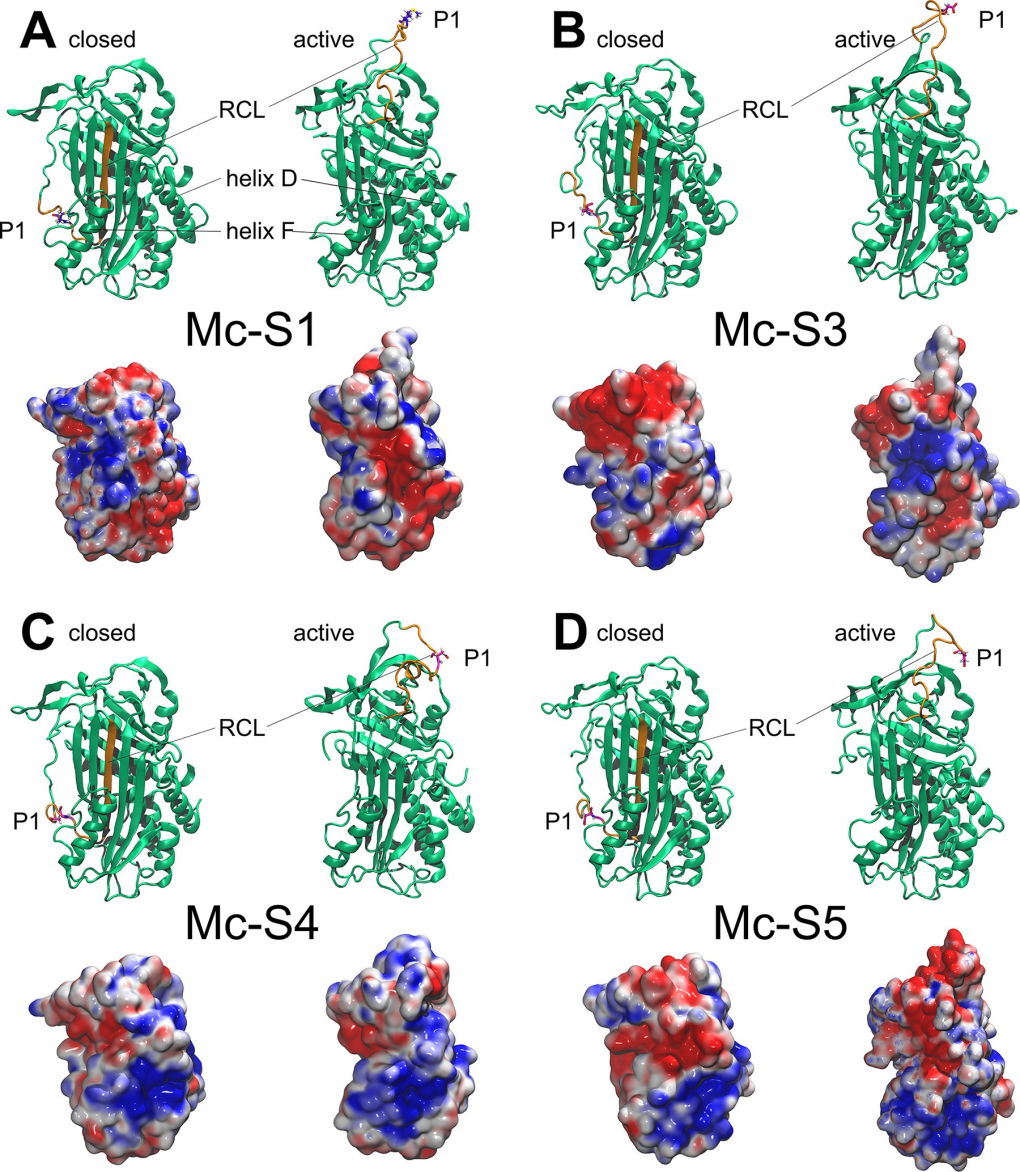
Ribbon and electrostatic surface models of serpins Mc-S1 (A), Mc-S3 (B), Mc-S4 (C) and Mc-S5 (D) of *Myxobolus cerebralis*. Both the closed (latent) and the active (native) conformations of proteins are presented. The reactive center loop (RCL) is labeled and highlighted in orange. The cleavage site in amino acid position P1 is in atomic thin tube representation. On the electrostatic surface models, red color represents regions with a potential value less than -2.0 kT; white color shows 0.0; blue shows regions greater than +2.0 kT.

In the course of the quality evaluation statistics of the generated serpin models, the Ramachandran plot values showed that approximately 99% of the ϕ, ψ angles of the polypeptide chain were in the allowed and generally allowed regions in all models, and only 0.5 to 1.2% were in the disallowed region (Table 5). The protein 3D structure quality control application, PROCESS, yielded an average quality rating for the model structures (3.5–5.5 on a scale of 1 to 10). The Z-scores of the generated serpin models calculated by ProSA were within the range of scores typically found for native proteins of similar size (-7.70 to -6.09) (Table 5). The aa sequence identity and similarity values of the templates 1IZ2, 2VDX, 1E03, 4KDS and 5CDZ used by I-TASSER compared to the studied serpins detected homology in most cases. The similarity between the aa sequences of the compared proteins was greater than 30% (39.9 to 52.1%), indicating that the modeled serpin structures are vastly similar to the templates used (S5 Table in S1 File). Although only minor structural differences were detected between the four serpin models, alterations were found in serpin Mc-S1 (Fig 4). The loop region between helix D and the first strand of the A sheet was two residues shorter in Mc-S1 than in the other serpins. In contrast, the loop between helix C and D was longer in Mc-S1 compared to the others (Table 4, Fig 4).

**Table 5:** Evaluation statistics of the protein models of *Myxobolus cerebralis* serpins, Mc-S1, Mc-S3, Mc-S4 and Mc-S5.

Model name	Form	Template serpin models	Ramachandran (%) (PROCHECK)	PROSESS overall quality	ProSA Z-score
Mc-S1	closed	1IZ2, 2VDX, 1E03, 4KDS	96.9^a^/2.3^b^/0.8^c^	4.5	-7.42
Mc-S1	active		96.0/3.1/0.9	3.5	-7.70
Mc-S3	closed	1IZ2, 5CDZ, 1E03, 2VDX	96.2/2.6/1.2	4.5	-6.09
Mc-S3	active		97.7/1.4/0.9	5.5	-6.76
Mc-S4	closed	1IZ2, 1E03, 5CDZ, 2VDX	96.5/2.6/0.9	4.5	-7.02
Mc-S4	active		96.0/2.9/1.1	3.5	-7.70
Mc-S5	closed	1IZ2, 1E03, 5CDZ, 2VDX	97.2/2.3/0.5	4.5	-6.22
Mc-S5	active		96.4/2.8/0.8	3.5	-6.54

^a^Allowed regions.

^b^Generously allowed regions.

^c^Disallowed regions.

Reference serpins used as template are labeled with their Protein Data Bank (PDB) accession number.

#### Function prediction

The protein secretion analyses using three applications showed that Mc-S1 is most likely secreted, whereas Mc-S3 is an intracellular protein. For Mc-S4 and Mc-S5, the analyses gave contradictory results, and protein secretion could not be predicted (Table 6). As an N-terminal signal protein was not detected in our study, putative secreted proteins were classified as unconventional protein secretions (UPS). High Arg content was observed in Mc-S3 that might have influenced the prediction of secretion using OutCyte. The isoelectric point (pI) of all serpins was slightly acidic, in a pH range between 5.18 and 6.11.

**Table 6:** Secretion of *Myxobolus cerebralis* serpin proteins predicted with models OutCyte, SectretomeP and SRTpred.

	OutCyte	Score (threshold = 0.5)	SecretomeP 2.0	Score (threshold = 0.5)	SRTpred	Score (threshold = 0.0)	Arginine content	pI
**Mc-S1**	UPS	**0.501**	UPS	**0.691**	Non-Secretory	**-0.450**	7	6.11
					UPS (AA)	**0.118**		
					UPS (properties)	**0.382**		
					UPS (dipeptide)	**0.208**		
**Mc-S3**	Intracellular	**0.668**	Intracellular	**0.485**	UPS (hybrid)	**0.945**	10	5.18
					UPS (AA)	**0.120**		
					UPS (properties)	**0.821**		
					Non-Secretory(dipeptide)	**-0.034**		
**Mc-S4**	UPS	**0.502**	Intracellular	**0.483**	UPS (hybrid)	**1.027**	6	6.05
					UPS (AA)	**0.145**		
					UPS (properties)	**0.625**		
					Non-Secretory(dipeptide)	**-0.610**		
**Mc-S5**	Intracellular	**0.514**	UPS/Intracellular	**0.527**	UPS (hybrid)	**1.125**	3	5.43
					UPS (AA)	**0.132**		
					UPS (properties)	**0.726**		
					UPS (dipeptide)	**0.790**		

pI: isoelectric point; UPS: unconventional protein secretion, i.e. secretory pathway that act without the involvement of an identified N-terminal signal peptide [70].

Based on the prediction of the ExPASy peptide cutter, Mc-S3, Mc-S4, and Mc-S5 are likely caspase inhibitors, as Asp is in P1, and non-polar aa with a long side-chain (i.e. Ile for Mc-S3) was found in position P4. On the other hand, Mc-S1 having Met in P1 is putatively a chymotrypsin- or elastase-like inhibitor. Electrostatic surface analysis of all modeled serpin structures showed that the protein surface around helix D is positively charged and thus, capable of binding the negatively charged heparin chain (Fig 4). *In silico* heparin docking modeled by ClusPro revealed that heparin was able to bind to the target region (i.e. helix D) of Mc-S3, Mc-S4 and Mc-S5 with low energy scores (-985.8 to -878.7 kcal/mol). For Mc-S1, the heparin docking was also possible, but the worse energy scores (-808.7 kcal/mol) and the lower number of heparin–serpin complex structure variants made the prediction of heparin binding uncertain (S6 Table in S1 File).

The inhibitory activity predicted with Glide showed two different functionalities among the serpins examined. The analysis confirmed that Mc-S1 is an anti-chymotrypsin, potentially with a chymotrypsin- or cathepsin-like target protease (S7 Table in S1 File). The models of Mc-S3, Mc-S4 and Mc-S5 were not able to dock properly to cathepsin G and chymotrypsin C targets. However, their docking to caspase-3 was successful and robust, strengthening the structural prediction that these serpins are indeed caspase-like inhibitors (S7 Table in S1 File).

## Discussion

The experimental exposures to *M*. *cerebralis* resulted in a high infection prevalence (100%) in both hosts, most likely due to the high infection dose (12,000 TAMs/fish for early stage, and 5,000 TAMs/fish for examining the late, sporogonic stage). The detected prevalence and intensity showed that doubling the number of samples for early-stage sampling was not absolutely necessary, although the larger number of samples definitely strengthened the statistical analysis. The reference genes GAPDH and RPL18 were chosen on the basis of previous findings on myxozoan gene expression profiling. It has been proven that GAPDH was the most uniformly expressed gene across the different developmental stages of *M*. *cerebralis* [55]. Although the suitability of RPL18 was not confirmed on the given species, it was used previously as a reliable reference gene for studying gene expression in *Tetracapsuloides bryosalmonae* causing proliferative kidney disease of salmonids [56, 57].

The obtained relative expression profiles of the examined four *M*. *cerebralis* serpins showed three different patterns. The serpin Mc-S1 was expressed at almost the same level at different stages of the life cycle, thus it is unlikely to play a significant role during host penetration and invasion. Mc-S3 and Mc-S5 were upregulated during fish invasion and early development, which is known to be a crucial step of parasite dissemination. During the first few hours following the penetration of *M*. *cerebralis* into fish, sporoplasms migrate between the epidermal cells. The sporoplasm aggregates disintegrate while forming smaller cell clusters, which are able to enter host cells and multiply intracellularly, and then they are released into the intercellular space. The single cell-doublets migrate deeper into the subcutis and, after a couple of days, they start their migration towards the place of spore formation in the cartilage of the skull and vertebrae [7, 8, 10, 14]. The relative expression of these serpins was significantly the lowest in the worm host, thus their expression profiles suggest that they may have a function during fish penetration.

Mc-S4 was significantly upregulated during fish invasion compared to the parasite’s migratory and sporogonic stages. Its relative expression was also high in *Tubifex tubifex*. Previous studies revealed that at this stage, the parasites develop intercellularly in the intestinal epithelium, the actinospore development takes about 90 days and comprising proliferation, gametogony and sporogony [11, 58]. Although a clear tendency could be observed in the changes of the Mc-S4 expression profile over the life cycle stages, differences were not significant among all stages examined.

The aa sequence analysis of the four examined serpins showed regular, serpin-like structure characteristics. However, the composition of the RCL region differed in several respects from that of the known (mainly human) serpins. Although the inhibitory serpins usually have Arg, Leu, or Lys in P1 position [22], three out of four serpins examined here had Asp–Gly in P1–P1’ positions, which suggests that the target protease of these serpins (Mc-S3, Mc-S4 and Mc-S5) is putatively a caspase-like protein. Besides Asp in P1, a large non-polar side chain in residue P4 of serpin is preferred by caspases (e.g. the viral serpin CrmA with Leu–VaI–Ala–Asp in P4–P1) [59]. Here, Mc-S3 fulfilled this requirement as long-chained, non-polar Ile was in P4, and Mc-S5 having Cys in P4 was most likely also favored. When comparing the RCL among myxozoan species, Asp in P1 occurred in the serpins of *M*. *cerebralis* and in those of closely related species, *M*. *squamalis* and *M*. *pendula*. A recent study revealed that not only these three parasite species but also their serpins were closely related at aa level [30]. However, the serpins of *Henneguya salminicola* and *Myxidium lieberkuehni* clustered in the same ‘genogroup’, while they had Gly or Arg in P1, thus the phylogenetic relation has probably no influence on functionality. The other representative of the cluster, serpin Mc-S1 that has Met in P1, seems to strengthen this conclusion. In contrast to myxozoan serpins, the dominance of putative inhibitory serpins such as antithrombin, antiplasmin (having Arg in P1) and antitrypsin (with Met in P1) was notable among free-living cnidarians.

Interestingly, the RCLs of some *M*. *cerebralis* serpins, especially that of Mc-S5, were rich in Cys. However, in the other myxozoan serpins, Cys content was relatively low, just like in the majority of known serpins. The serpins, especially extracellular ones, are usually not rich in Cys, mainly because of the higher chance of transient disulfide bond formation that may complicate secretion [22, 60]. The secretion potential of serpins was also studied here. Secretion may be a crucial factor for serpins having inhibitory and/or immunomodulatory activity. The predictions of protein localization using OutCyte showed that Mc-S1 is most likely secreted, Mc-S3 is intracellular, whereas the analysis of Mc-S4 and Mc-S5 yielded ambiguous results. OutCyte is a fast and accurate tool for the prediction of unconventional protein secretion (UPS) [48]. One of the core parameters of the analysis is that secretion is not preferred for proteins with high Arg content. Maybe that was the reason why Mc-S3 with high Arg content was defined as intracellular.

Residues P12–P9 in inhibitory serpins show >50% conservation of Ala at each position, whereas non-inhibitory serpins have few or no Ala at these positions [22]. The high Ala content in the examined serpins suggests that they do have inhibitory activity. The isoelectric point (pI) of the examined serpins was between pH 5.18 and 6.11, suggesting that the parasite’s serpins are most likely solubilized in slightly alkaline tissues (such as blood) of the fish hosts (pH 7.3–7.6). Physicochemical analysis of the recombinant proteins would be able to clarify whether the examined serpins are secreted.

Extracellular serpins generally inhibit serine proteases belonging to the chymotrypsin family. Intracellular serpins, on the other hand, target cysteine proteases (mostly caspases) [61]. Several extracellular serpins, mainly found in vertebrate blood plasma, are activated as inhibitors of target proteinases by binding to heparin or other linear, negatively charged glycosaminoglycans [22]. These include serpins involved in the regulation of proteinases of the blood coagulation and fibrinolysis systems (e.g. antithrombin, heparin cofactor II, protein C inhibitor, protease nexin 1, etc.). Heparin can act as a co-factor for serpins, accelerating and/or improving the inhibition of proteases; for example the affinity of α-1-antitrypsin to trypsin increases significantly in the presence of heparin [62, 63]. The formation of the serpin–heparin–protease complex is a known phenomenon also in parasites. Serpins FhS-2 and FhS-4 of *Fasciola hepatica* are able to bind heparin with a strong affinity, and they inhibit pro-inflammatory and pro-coagulant proteases [64]. The present study suggests that all four serpins are able to bind heparin. However, we do not know yet whether they form such ‘heparin-mediated’ complexes. The prediction of putative target proteases has revealed that Mc-S1 is a chymotrypsin-like inhibitor, whereas Mc-S3, Mc-S4 and Mc-S5 are caspase-like inhibitors. The targets of antichymotrypsins are usually elastase, cathepsin G, or chymase proteases [22]. Mc-S1 with P1-residue Met seems to be able to bind chymotrypsin C and cathepsin G. Cathepsin G may play an important role in anti-inflammatory responses by eliminating intracellular pathogens and by disintegrating tissues at the sites of inflammation [65]. On the basis of the present findings, it is possible that Mc-S1 takes part in the inactivation of cathepsin G or similar host proteases. The existing knowledge on the function of myxozoan protease inhibitors is rather scarce. Although the function of myxozoan serpins has not been analyzed up to now, a recent study has presented the functional analysis of a member of another large protease family, the cysteine protease inhibitors [66]. The characterization of a secretory cystatin from *Thelohanellus kitauei* showed that TK-cystatin was able to inhibit cathepsin L, indicating that the inhibitor may have immune-suppressive activity.

Our functional predictions show that the other three *M*. *cerebralis* serpins, Mc-S3, Mc-S4 and Mc-S5, are most likely caspase inhibitors. Caspases are the key effector molecules of apoptosis, the physiological cell death process, although some of them are involved in the activation of cytokines. Active caspases can be controlled by a variety of inhibitors including serpins [67, 68]. Serpins implicated in the regulation of cell death include the viral proteins CrmA and SPI-1, the granzyme B inhibitor, PI-9, etc.. Another endogenous serpin, PN-I, prevents the delivery of an apoptotic signal by inhibiting an extracellular proteinase from cleaving a cell surface receptor [59]. The putative function of an anti-caspase serpin in myxozoan parasites is not known yet. A previous study reported that a ubiquitin-conjugating enzyme of *M*. *cerebralis* was involved in the host recognition of the parasite, and its relative expression was significantly upregulated during the first few hours of fish invasion [20]. Some caspase inhibitors (known as inhibitors of apoptosis proteins) use their ubiquitination properties to subject caspases to proteasomal degradation [69]. As the degradation of primary cell aggregates and other lytic processes occur in the first few hours of host invasion, the involvement of the three *M*. *cerebralis* serpins in the protein or even parasite cell degradation cannot be excluded.

## Conclusions

The inactivation of the parasite at an early stage would interrupt its life cycle in fish, and thereby it would prevent the development of whirling disease. The expression profile and functional prediction of serpins Mc-S3 and Mc-S5 suggest that these caspase-like protease inhibitors are promising targets for this purpose. Nevertheless, Mc-S1 as a putative antichymotrypsin should not be excluded from further functional analysis either, taking into account the anti-inflammatory potential of this putatively extracellular protease inhibitor. Therefore, further studies on recombinant proteins are planned to elucidate the functional characteristics (e.g. inhibitory activity) of the selected serpins in *in vitro* and *in vivo* assays.

## Supporting information

S1 Fig Relative expression profiles of serpin genes (A) Mc-S1, (B) Mc-S3, (C) Mc-S4, (D) Mc-S5 at different time points of *Myxobolus cerebralis* development. Log10 transformed, calibrated normalized relative quantity (log10 CNRQ) values were normalized to reference gene **GAPDH**. Significant differences indicated with uppercase letters (*p* < 0.001) or lowercase letters (*p* < 0.01). The same letter was given when the difference was significant between two groups.
(TIF)

S1 Table The efficiencies of *M*. *cerebralis* serpin-specific qPCR assays, including reference genes GAPDH and RPL18 (A), and GAPDH only (B).
(ZIP)

S2 Table Raw Cq values and CNRQ calculations of *M*. *cerebralis* serpin-specific qPCR assays, including reference genes GAPDH and RPL18 (A), and GAPDH only (B). n.d. no data; *: valid Cq value was converted to unknown CNRQ data when one of the reference genes (here RPL18) gave negative result.
(XLSX)

S1 File (DOCX)
