# Supplementary figures and images for: Serine protease inhibitors of the whirling disease parasite Myxobolus cerebralis (Cnidaria, Myxozoa): Expression profiling and functional predictions
Source: PLoS One. 2021 Mar 29;16(3):e0249266. doi: 10.1371/journal.pone.0249266 (PMC8007001; doi:10.1371/journal.pone.0249266)

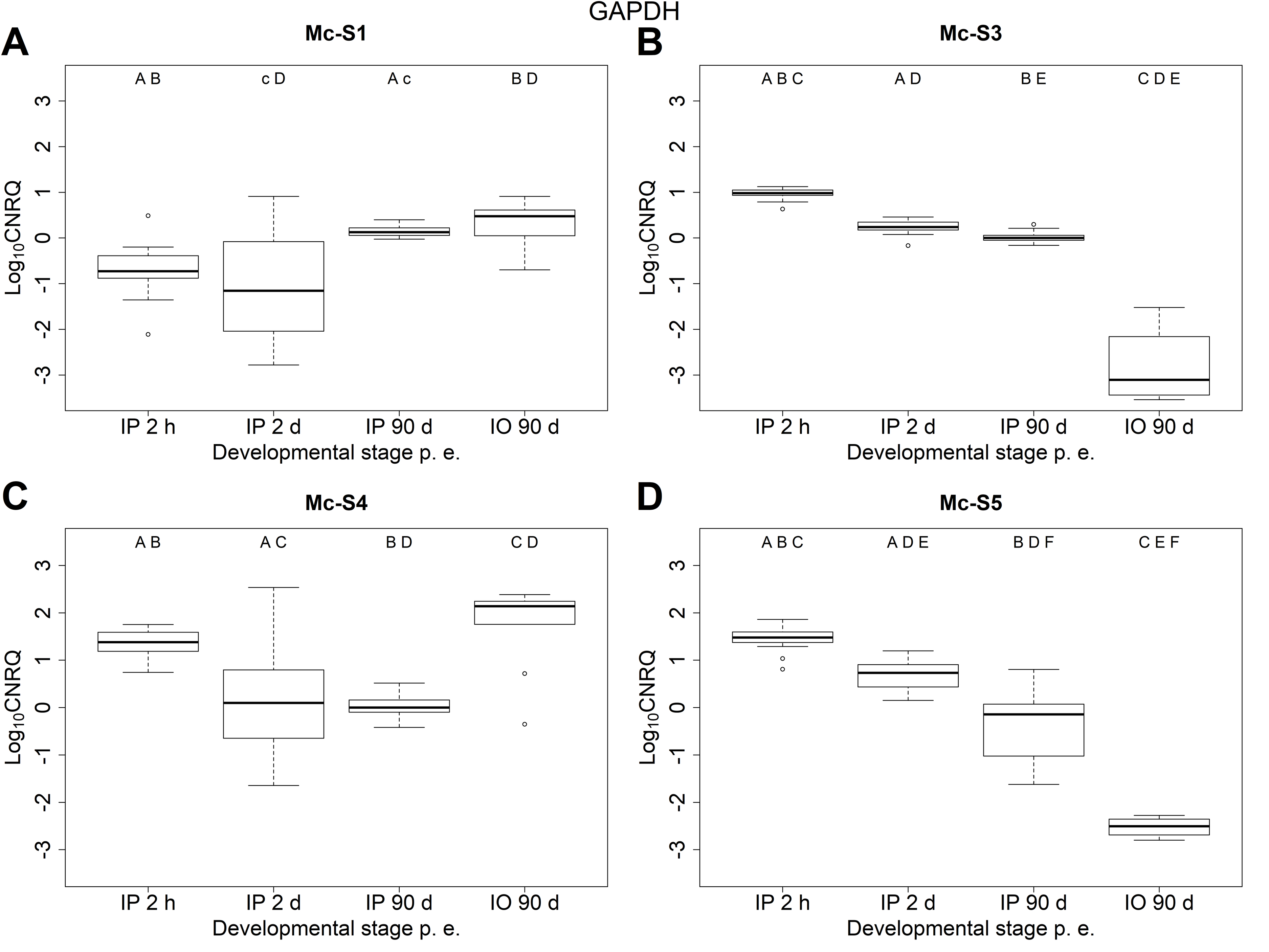

Supplement: S1 Fig — Relative expression profiles of serpin genes (A) Mc-S1, (B) Mc-S3, (C) Mc-S4, (D) Mc-S5 at different time points of Myxobolus cerebralis development. Log10 transformed, calibrated normalized relative quantity (log10 CNRQ) values were normalized to reference gene GAPDH. Significant differences indicated with uppercase letters (p < 0.001) or lowercase letters (p < 0.01). The same letter was given when the difference was significant between two groups. (TIF) [file pone.0249266.s001.tif]
