# Supplementary material for: Serine protease inhibitors of the whirling disease parasite Myxobolus cerebralis (Cnidaria, Myxozoa): Expression profiling and functional predictions
Source: PLoS One. 2021 Mar 29;16(3):e0249266. doi: 10.1371/journal.pone.0249266 (PMC8007001; doi:10.1371/journal.pone.0249266)
Supplement: S1 File — (DOCX) [file pone.0249266.s004.docx]

**Eszterbauer et al.** Serine protease inhibitors of the whirling disease parasite *Myxobolus cerebralis* (Cnidaria, Myxozoa): Expression profiling and functional predictions

**S3 Table.** Descriptive statistics of the relative expression of *Myxobolus cerebralis* serpins (Mc-S1, Mc-S3, Mc-S4 and Mc-S5) in four different developmental stages on the basis of log-transformed, calibrated, normalized, relative quantities (Log10 CNRQ). Values were normalized to GAPDH reference gene. Minimum and maximum intensity (Min – Max); standard deviation (SD); number of positive/total samples (N_+_/N_t_). IP: intrapiscine, IO: intraoligochaete developmental stage.

| Serpin name | Developmental stage | **log_10_CNRQ** | | | **N_+_/N_t_** |
| --- | --- | --- | --- | --- | --- |
|  |  | Min – Max | Median | SD |  |
| Mc-S1 | IP 2 h p.e. | -2.1123 – 0.4864 | -0.7286 | 0.5846 | 15/19 |
|  | IP 2 d p.e. | -2.7788 – 0.9091 | -1.1563 | 1.4794 | 5/20 |
|  | IP 90 d p.e. | -0.0252 – 0.3976 | 0.1268 | 0.1335 | 9/9 |
|  | IO 90 d p.e. | -0.6964 – 0.9091 | 0.4777 | 0.5447 | 9/9 |
| Mc-S3 | IP 2 h p.e. | 0.6321 – 1.1248 | 0.9834 | 0.1200 | 19/19 |
|  | IP 2 d p.e. | -0.1691 – 0.4588 | 0.2397 | 0.1410 | 20/20 |
|  | IP 90 d p.e. | -0.1599 – 0.2967 | 0.0000 | 0.1431 | 9/9 |
|  | IO 90 d p.e. | -3.5403 – -1.5216 | -3.1044 | 0.7983 | 6/9 |
| Mc-S4 | IP 2 h p.e. | 0.7427 – 1.7527 | 1.3795 | 0.2924 | 19/19 |
|  | IP 2 d p.e. | -1.6459 – 2.5367 | 0.0988 | 1.0979 | 16/20 |
|  | IP 90 d p.e. | -0.4183 – 0.5157 | 0.0000 | 0.2652 | 9/9 |
|  | IO 90 d p.e. | -0.3540 – 2.3852 | 2.1402 | 0.9347 | 9/9 |
| Mc-S5 | IP 2 h p.e. | 0.8098 – 1.8621 | 1.4793 | 0.2440 | 19/19 |
|  | IP 2 d p.e. | 0.1501 – 1.1970 | 0.7345 | 0.3000 | 20/20 |
|  | IP 90 d p.e. | -1.6207 – 0.8062 | -0.1426 | 0.7904 | 8/9 |
|  | IO 90 d p.e. | -2.7994 – -2.2746 | -2.5052 | 0.2231 | 4/9 |

**S4 Table.** P-values of ANOVA and Tukey’s post-hoc tests on log-transformed, calibrated, normalized, relative quantities (Log10 CNRQ) obtained with measuring the relative expression of serpins (Mc-S1, Mc-S3, Mc-S4 and Mc-S5) in four different developmental stages of *Myxobolus cerebralis*. Reference gene: GAPDH. IP: intrapiscine, IO: intraoligochaete developmental stage.

| Tukey’s test | **Mc-S1** | **Mc-S3** | **Mc-S4** | **Mc-S5** |
| --- | --- | --- | --- | --- |
| IP 2 d – IP 2 h | 0.77580 | <1e-04 | <0.001 | 1.08e-06 |
| IP 90 d – IP 2 h | 0.03123 | <1e-04 | <0.001 | < 1e-06 |
| IO 90 d – IP 2 h | 0.00892 | <1e-04 | 0.570 | < 1e-06 |
| IP 90 d – IP 2 d | 0.02052 | 0.219 | 0.998 | < 1e-06 |
| IO 90 d – IP 2 d | 0.00763 | <1e-04 | <0.001 | < 1e-06 |
| IO 90 d – IP 90 d | 0.96961 | <1e-04 | <0.001 | < 1e-06 |
| ANOVA | 0.0008725 | < 2.2e-16 | 1.937e-07 | < 2.2e-16 |

**S5 Table.** Amino acid sequence identity and similarity values from pairwise protein sequence alignments of *Myxobolus cerebralis* serpins. Reference serpins used as templates are labeled with their Protein Data Bank (PDB) accession number.

| Templates | Mc-S1 Id% | Mc-S1 Sim% | Mc-S3 Id% | Mc-S3 Sim% | Mc-S4 Id% | Mc-S4 Sim% | Mc-S5 Id% | Mc-S5 Sim% |
| --- | --- | --- | --- | --- | --- | --- | --- | --- |
| 1IZ2 | 24.5 | 44.7 | 26.0 | 45.3 | 24.1 | 44.3 | 24.9 | 43.7 |
| 2VDX | 26.6 | 49.0 | 27.0 | 45.0 | 26.2 | 44.5 | 24.9 | 45.5 |
| 1E03 | 25.1 | 42.8 | 23.6 | 39.9 | 24.7 | 40.4 | 21.3 | 36.0 |
| 4KDS | 23.5 | 45.0 | 26.0 | 46.8 | 26.3 | 44.9 | 24.8 | 44.1 |
| 5CDZ | 30.2 | 52.1 | 29.9 | 49.4 | 31.8 | 50.6 | 29.2 | 48.2 |

**S6 Table.** Evaluation of heparin docking modeling on the examined serpins of *Myxobolus cerebralis*.

| Serpins | Heparin binding around helix D | No. of the best clusters / No. of all hits | Lowest binding energy (kcal/mol) |
| --- | --- | --- | --- |
| Mc-S1 | Yes | 525 / 1799 | -808.7 |
| Mc-S3 | Yes | 406 / 1800 | -878.4 |
| Mc-S4 | Yes | 441 / 1800 | -985.8 |
| Mc-S5 | Yes | 571 / 1800 | -925.8 |

**S7 Table.** Evaluation of the serpin RCL octamer peptide (P4–P4’) docking to the target protease active site.

|  | Docking score | Glide gscore | Glide emodel / (kcal/mol) | Does P1 side chain dock to the active site of binding pocket? |
| --- | --- | --- | --- | --- |
| **Caspase3** (5JFT) | |  |  |  |
| Mc-S1 | -7.598 | -10.278 | -159.612 | No |
| Mc-S3 | -9.385 | -10.556 | -155.484 | Yes |
| Mc-S4 | -11.483 | -11.871 | -191.261 | Yes |
| Mc-S5 | -9.982 | -10.458 | -152.941 | Yes |
| **Chymotrypsin C** (4H4F) | |  |  |  |
| Mc-S1 | -10.689 | -11.700 | -152.739 | Yes |
| Mc-S3 | -7.414 | -9.984 | -156.243 | No |
| Mc-S4 | -8.445 | -10.542 | -176.319 | No |
| Mc-S5 | -10.419 | -10.895 | -157.622 | No |
| **Cathepsin G** (1CGH) | |  |  |  |
| Mc-S1 | -9.350 | -9.476 | -112.652 | Yes |
| Mc-S3 | -8.199 | -9.370 | -129.702 | No |
| Mc-S4 | -9.134 | -9.162 | -142.445 | No |
| Mc-S5 | -9.462 | -9.863 | -130.014 | No |
